# Sodium Alginate-Quaternary Polymethacrylate Composites: Characterization of Dispersions and Calcium Ion Cross-Linked Gel Beads

**DOI:** 10.3390/gels8110739

**Published:** 2022-11-15

**Authors:** Wanwisa Khunawattanakul, Napaphak Jaipakdee, Thitiphorn Rongthong, Narin Chansri, Pathomthat Srisuk, Padungkwan Chitropas, Thaned Pongjanyakul

**Affiliations:** 1 Division of Pharmaceutical Sciences, Faculty of Pharmacy, Mahasarakham University, Maha Sarakham 44150, Thailand; 2 Division of Pharmaceutical Technology, Faculty of Pharmaceutical Sciences, Khon Kaen University, Khon Kaen 40002, Thailand; 3 Chulabhorn Royal Pharmaceutical Manufacturing Facility, Chulabhorn Royal Academy, Bangkok 10210, Thailand

**Keywords:** sodium alginate, quaternary polymethacrylate, calcium alginate beads, composite dispersion, viscosity enhancement, bead strength, drug release

## Abstract

The objective of this work was to examine the effect of quaternary polymethacrylate (QPM), a water-insoluble polymer with a positive charge, on the characteristics of the sodium alginate (SA) dispersions and the calcium alginate (CA) gel beads containing propranolol HCl (PPN). The SA-QPM composite dispersions presented the formation of flocculates with a negative charge due to the electrostatic interaction of both substances. The QPM addition did not affect the SA dispersions’ Newtonian flow, but the composite dispersions’ viscosity enhancement was found. The PPN-loaded CA-QPM gel beads had more spherical than the PPN-loaded CA gel beads. The incorporation of QPM caused a bigger particle size, higher drug entrapment efficiency, and greater particle strength of the gel beads. Despite the similar water uptake property, the PPN-loaded CA-QPM gel beads displayed lower burst release and slower drug release rate than the PPN-loaded CA gel beads. However, the drug release from the PPN-loaded CA-QPM gel beads involved drug diffusion and matrix swelling mechanisms. This study demonstrated that adding QPM into the SA dispersions leads to a viscosity synergism. The CA-QPM gel beads display a good potential for use as a bioactive compound delivery system.

## 1. Introduction

Sodium alginate (SA), a sodium salt of alginic acid, is a natural polysaccharide from marine brown algae. SA has been employed as an additive in foods and pharmaceutical dosage forms [1,2]. SA consists of two uronic acids, namely α-L-guluronic and β-D-mannuronic acids. The arrangement of both uronic acids is homopolymeric blocks and blocks with an alternating sequence [2]. SA possesses good hydration and swelling when exposed to water. The outstanding gelation property of SA is created by cross-linking the uronic acids with divalent cations, such as calcium ions. The gelation of SA with calcium ions is known as the ‘egg box’ structure [3]. The primary mechanism of this gelation implicates the chain sequences that adapt a regular two-fold conformation and dimerize with calcium ion chelation. Each calcium ion has involved nine coordination links with an oxygen atom, leading to a three-dimensional network of calcium alginate (CA).

The calcium ion gelation of SA has been widely applied for preparing a CA gel bead for employment as a drug delivery system by dropping the SA dispersion with drugs into a calcium chloride bath [4,5]. This method is called ionotropic gelation [6]. The CA gel beads can protect an acid-labile drug from the stomach condition, and the drug was consequently liberated from the gel beads in the small intestine condition [7,8]. Additionally, this delivery system is also applied to produce artificial foods, such as fish roe [9], and to encapsulate bioactive compounds, such as nutraceuticals [10], antioxidants [11], antimicrobials [12], and flavors [13].

Adding both water-soluble and water-insoluble substances can modify the physical properties of the CA gel beads. Water-soluble substances, such as hydrophilic macromolecules [14,15,16,17,18,19,20], can increase drug entrapment efficiency (DEE), swelling property, and drug release of the CA gel beads. The alternative approach for modulating the CA gel bead properties is the incorporation of water-insoluble substances. These substances, such as wax [21,22] and clays [23,24,25], are used, which can enhance DEE and retard drug release from the gel beads due to an increase in hydrophobic property and molecular interaction of silanol groups of clay with carboxyl groups of SA, respectively. Chitin, a water-insoluble polymer, is added to the CA gel beads to retard drug release in a neutral condition of the small intestine owing to the complexation between the carboxyl groups of SA and the amino groups of chitin [26].

Quaternary polymethacrylate (QPM), a water-insoluble copolymer, is synthesized from acrylic and methacrylic esters. This copolymer contains positively charged quaternary ammonium groups that have chloride ions as counter ions in their structures. QPM in aqueous dispersion form is available as the market products, such as Eudragit^®^ RS30D and Eudragit^®^ RL30D. Both products contain 5 and 10% quaternary ammonium groups [27]. Therefore, it has been employed as a material for film coating in pharmaceuticals [27]. However, the QPM films present poor mechanical properties that can be modified by the addition of water-soluble or water-insoluble plasticizers [28]. Furthermore, anionic hydrophilic polymers, such as pectin [29], which interact with QPM via electrostatic force, can decrease the hydration and swelling of QPM films, resulting in the retardation of drug diffusivity through the film. Recently, SA dispersion was able to blend into the QPM dispersion with different ratios, and the molecular interactions between them via electrostatic force can induce the flocculate formation in the composite dispersions [30]. The QPM films blended with SA displayed a decisive shift of the COO^−^ (asymmetric and symmetric) stretching peak of SA, which was revealed via FTIR spectroscopy [30], indicative of an ionic interaction of the carboxyl groups of SA with quaternary ammonium groups of QPM. This phenomenon causes a change in physicochemical properties and an alteration of drug permeability across the films. Therefore, it is interesting to blend QPM dispersion into the SA dispersions and prepare the CA-QPM gel beads via ionotropic gelation using calcium ions. As a result, the QPM may cause a characteristic change in the SA dispersions and the CA gel beads.

This work aimed to investigate the effect of QPM on the characteristics of the SA dispersions and the CA gel beads. The composite dispersions were prepared using different SA:QPM ratios and the particle size and zeta potential of the dispersed phase and rheological properties of the composite dispersions were characterized. Moreover, the drug-loaded CA-QPM gel beads were prepared using ionotropic gelation with calcium ions. Propranolol HCl (PPN) was used as a model drug in this study. The gel bead properties, such as particle size, DEE, particle strength, water uptake, and drug release, were examined. The CA-QPM gel beads obtained may potentially be used as a delivery system for bioactive compounds in foods and pharmaceuticals.

## 2. Results and Discussion

### 2.1. Appearance of SA-QPM Composite Dispersions

**Figure 1:**
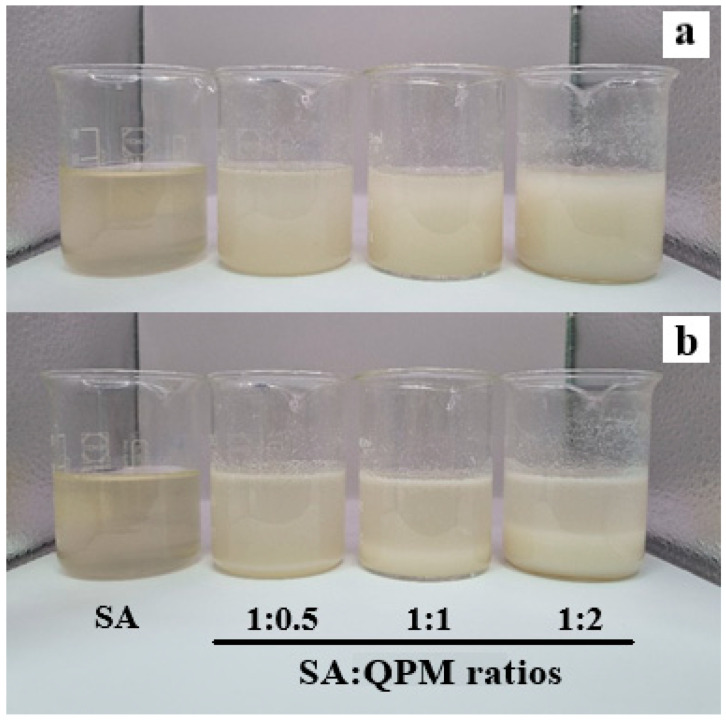
Appearance of SA and SA-QPM dispersions after preparation (**a**) and storing at room temperature for 24 h (**b**).

**Figure 2:**
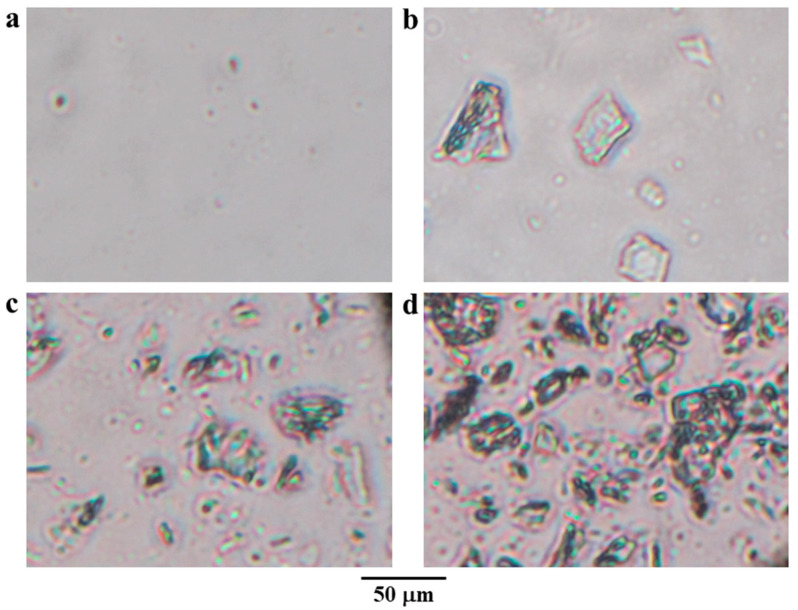
Microscopic morphology of QPM particles in QPM dispersion (**a**), and SA-QPM dispersions at the ratios of 1:0.5 (**b**), 1:1 (**c**), and 1:2 (**d**).

SA dispersion was a clear and viscous liquid, whereas QPM dispersion was a milky liquid. Therefore, the QPM dispersion could easily be mixed into the SA dispersion, and the SA-QPM composite dispersions showed more turbidity after preparations when increasing QPM content, as shown in Figure 1a. The sedimentation of the dispersed phase in the SA-QPM composite dispersions was initially observed when left at room temperature for 4 h. The appearance of all dispersions stored for 24 h is displayed in Figure 1b. It can be seen that increasing QPM content resulted in high sedimentation volume in the composite dispersions. The microscopic morphology of the QPM particles in the composite dispersions after 24 h storage was observed using inverted microscopy, which is shown in Figure 2. Figure 2a shows the dispersed phase in the QPM dispersion that it was complicated to observe and view the picture because the QPM particles were tiny and fall in nanosized particles [31]. However, the flocculation of QPM particles could be observed in the SA-QPM composite dispersions, and these flocculate amounts increased with increased QPM content added (Figure 2b–d). These results suggested that QPM particles could form the flocculates by SA molecules. The SA-QPM flocculation could be induced by electrostatic interaction between QPM particles and SA molecules after simple mixing.

### 2.2. Characteristics of the SA-QPM Composite Dispersions

**Figure 3:**
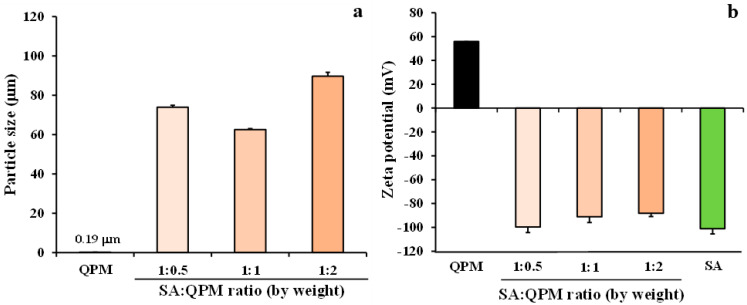
Particle size (**a**) and zeta potential (**b**) of dispersed phase in QPM, SA, and SA-QPM dispersions. Each value is the mean ± S.D., *n* = 3.

The particle size and zeta potential of the dispersed phase of the SA-QPM composite dispersions are presented in Figure 3. The particle size of QPM dispersion was determined to be 0.19 μm, whereas the SA dispersion could not be measured due to its very small size in a colloid level. The SA-QPM flocculates size of the composite dispersion was over the range of 62.6–89.7 μm, in which the SA-QPM composite dispersion in the ratio of 1:2 gave the greatest flocculate size (Figure 3a). The zeta potential values of all dispersions are presented in Figure 3b. The zeta potential values of QPM and SA dispersions were 55.9 ± 0.04 and −101.2 ± 4.31 mV (*n* = 3), respectively. The opposite charge of both substances brought about an electrostatic interaction after simple mixing, leading to a flocculate formation. The SA-QPM flocculates still showed a negative charge, although the content of QPM by weight was 2-fold higher than the SA content in the composite dispersion. However, the zeta potential of the SA-QPM composite dispersions decreased with increasing QPM ratios. This result suggested that the positively charged QPM particles could not neutralize the negative charge of SA molecules. In contrast with the previous study, the SA amount at 12.5% based on QPM weight was added to the QPM dispersion. As a result, the zeta potential value of the dispersed phase of the composite dispersion was changed from positive to negative charges, indicating that a small amount of SA could be completely adsorbed onto the QPM particles [30]. Therefore, it could be described in this study that QPM particles could be adsorbed and covered by the long-chain molecules of SA to form the flocculates, leading to a negatively charged surface. Additionally, this phenomenon was similar to the case of chitosan–clay dispersions; the flocculates showed a positive charge of polymeric chitosan even though the negatively charged clay was blended in higher content than chitosan [32,33].

**Figure 4:**
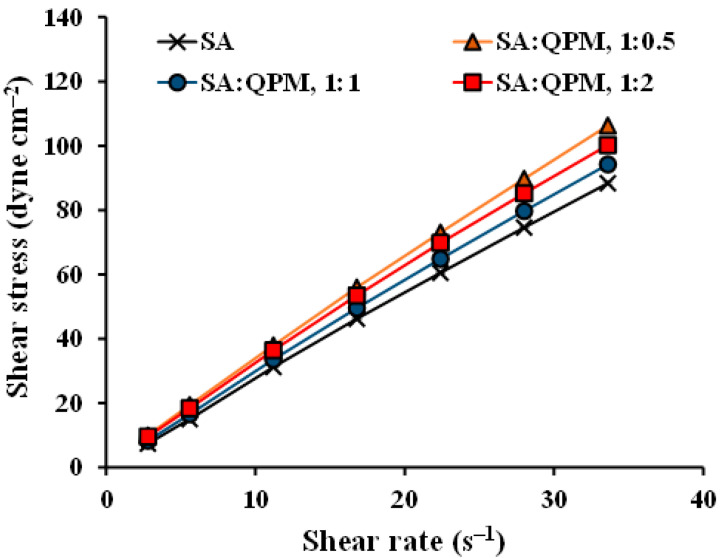
Rheograms of SA and SA-QPM dispersions. Each value is the mean ± S.D., *n* = 3.

The rheological properties and viscosity of the SA-QPM composite dispersion were also characterized in this study. The relationship between shear stress and shear rate (rheogram or flow curve) is illustrated in Figure 4. It could be seen that the rheogram of all dispersions seemed to present a linear line. Therefore, the exponential formula (log *G* = *N* log *F*−log *η*) was applied to analyze flow type. The exponential constant (*N*) and viscosity coefficient (*η*) computed for all dispersions are listed in Table 1. The *N* value of the SA dispersions was 1.00 ± 0.01 (*n* = 3), indicative of Newtonian flow. This result was similar to the previous report [34,35] that the Newtonian flow of SA dispersion was changed to pseudoplastic flow when the SA concentration was equal to or higher than 2.5 %*w*/*v* [35]. Furthermore, the SA-QPM composite dispersions provided *N* values in the range of 0.94–0.98, suggesting that the composite dispersions still had the Newtonian flow after molecular interaction between SA and QPM occurred. The composite dispersions’ viscosity coefficient (*η*) values were remarkably higher than that of the SA dispersion. However, the η values could not be used for comparison because they were obtained from the different N values. Therefore, the viscosity at 22.4 s^−1^ shear rate of the dispersions was used, and these values are listed in Table 1. The viscosity at 22.4 s^−1^ shear rate of the SA dispersion was 270.4 cP, and the viscosity of the composite dispersions was greater than that of the SA dispersion. However, a viscosity increase in the composite dispersions was not correlated to the contents of QPM incorporated. Generally, the SA dispersion presented a higher viscosity with increasing clay content added [23]. However, there was a case of polymethacrylate-silica composite dispersions that an increase in viscosity of the composite dispersion was not related to the silica added. However, the incorporation of silica brought about a higher viscosity of the composite dispersion [36]. This outcome could have occurred in the rheological properties of the composite dispersions. Therefore, this study showed that molecular interaction between SA and QPM brought about the formation of numerous points of contact and a small three-dimensional network, leading to a viscosity enhancement of the composite dispersions. However, these interactions did not influence the flow type of the SA dispersions.

### 2.3. Particle Morphology and DEE of PPN-Loaded CA-QPM Gel Beads

**Figure 5:**
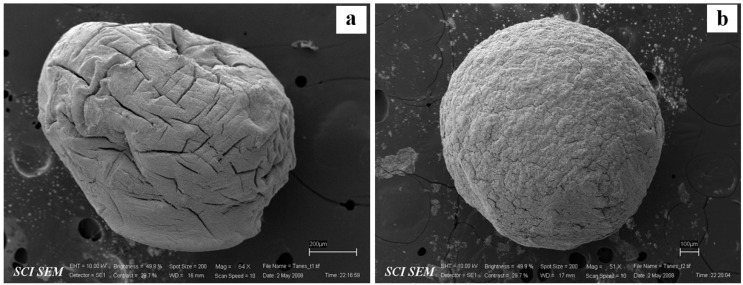
Particle morphology of PPN-loaded CA gel bead (**a**) and PPN-loaded CA-QPM gel bead at the SA:QPM ratios of 1:2 (**b**).

The SA dispersions incorporated with PPN were dropped into the calcium chloride solution to form CA gel beads. The CA-QPM gel beads using different SA:QPM ratios were also prepared. The particle size of all gel beads is listed in Table 2. The PPN-loaded CA gel beads provided the smallest size (1.03 ± 0.12 mm) compared to the PPN-loaded CA-QPM gel beads. An increase in QPM ratios led to a bigger particle size of the CA-QPM gel beads. This result is caused by the increasing solid content in the dispersion before cross-linking. This finding was in agreement with the previous reports that incorporated some water-insoluble substances into the CA gel beads [23,25,37]. The particle morphology of the PPN-loaded CA gel beads presented a spherical shape with a collapsed region (Figure 5a) caused by the shrinkage of the wet gel beads during the drying process. On the other hand, the sphere particle was found in the PPN-loaded CA-QPM gel beads at the SA:QPM ratio of 1:2 (Figure 5b). It could be explained that the addition of QPM (small solid particles) could prevent shrinkage and stabilize a bead shape during drying.

The PPN content in the gel beads decreased with increasing QPM ratios (Table 2). This result was due to the increased solid content in the CA gel beads when incorporating QPM. However, the addition of QPM increased DEE in the gel beads (Table 2). In the case of the PPN-loaded CA beads, the drug loss was caused by water leakage from the wet gel beads during the cross-linking process in the preparation period [38]. PPN is a water-soluble drug, and the water leakage strongly affected the drug diffusion out of the gel beads. However, in molecular interaction between SA and PPN in the CA gel beads, it was previously reported that the hydroxyl and carboxyl (asymmetric and symmetric) stretching peaks of the CA gel beads were moved to a lower wavenumber when incorporating with PPN, which was investigated by FTIR spectroscopy [25]. This interaction could promote an entrapment of PPN in the gel bead’s structure and decrease the loss of PPN from the gel bead. The incorporation of QPM into the CA gel beads caused an increasing barrier preventing water leakage from the wet gel beads. Moreover, molecular interaction between SA and QPM could create a denser network matrix and higher tortuosity in the wet gel beads, leading to a retardation of PPN diffusion into the surrounding medium during the calcium ion cross-linking process. Additionally, the greater particle size of the CA-QPM gel beads had a longer path length for drug diffusion. Thus, the reduction in PPN loss from the CA-QPM gel beads was obtained.

### 2.4. Particle Strength of PPN-Loaded CA-QPM Gel Beads

**Figure 6:**
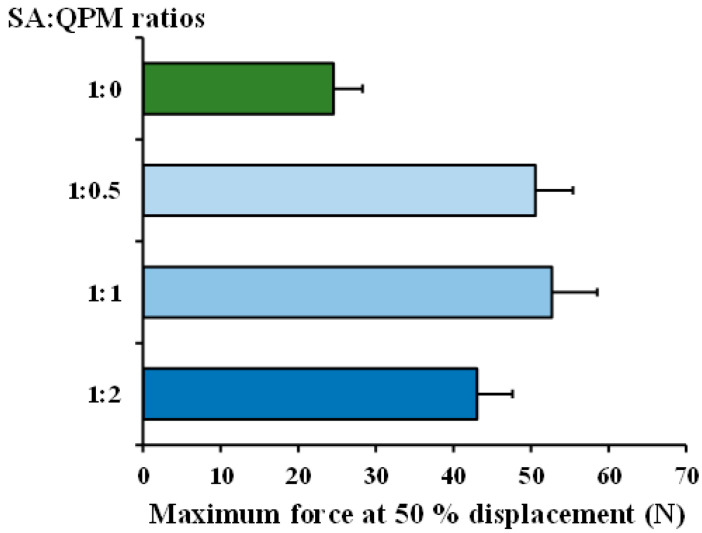
Mechanical property of PPN-loaded CA-QPM gel beads at different SA:QPM ratios. Each value is the mean ± S.D., *n* = 10.

Effect of QPM ratios on the maximum force at 50% displacement of the CA-QPM gel beads are displayed in Figure 6. These maximum forces represented the particle strength of the gel beads. The PPN-loaded CA gel beads presented the lowest value of the maximum force at 50% displacement, indicative of the weakest gel bead. However, incorporating QPM in the gel beads resulted in a pronounced increase in these maximum forces. This result suggested that the interaction of SA and QPM could create a dense matrix structure, and the embedded QPM particles could reinforce the particle strength of the gel beads. However, a slight decrease in the particle strength was obtained in the PPN-loaded CA-QPM gel beads at the SA:QPM ratio of 1:2. It could be explained that the highest QPM content added could disturb the calcium ions cross-linking of the SA molecules in the gel beads, leading to an incomplete cross-linking in the interior or some regions of the gel beads.

### 2.5. Water Uptake and Drug Release of PPN-Loaded CA-QPM Gel Beads

**Figure 7:**
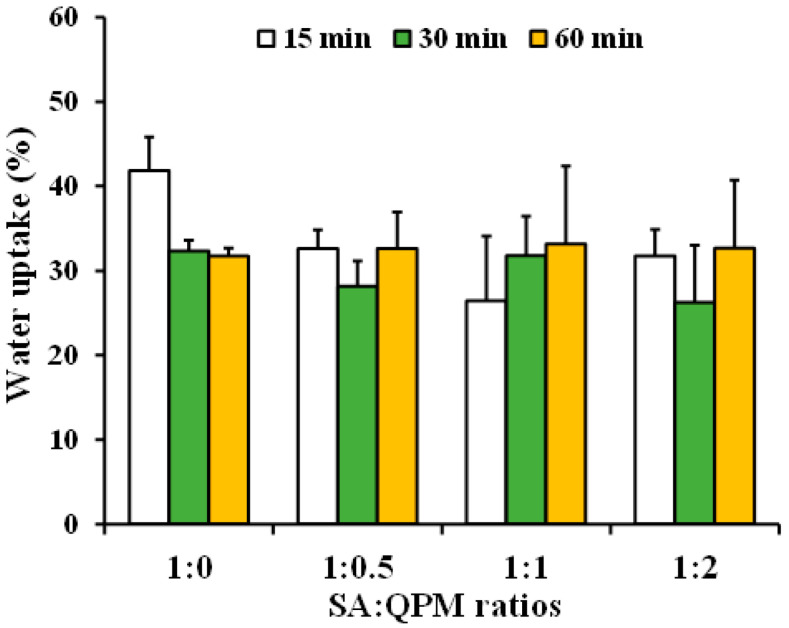
Water uptake of PPN-loaded CA-QPM gel beads at different SA:QPM ratios. Each value is the mean ± S.D., *n* = 3.

**Figure 8:**
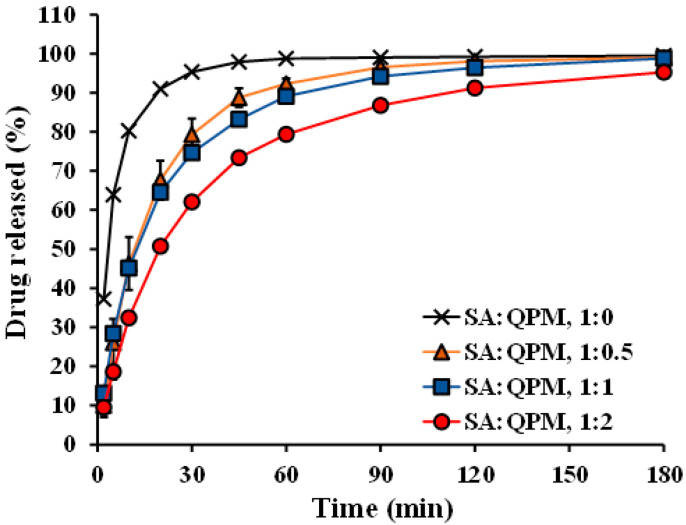
PPN release profiles of PPN-loaded CA-QPM gel beads at different SA:QPM ratios. Each value is the mean ± S.D., *n* = 3.

The water uptake and drug release of the PPN-loaded CA-QPM gel beads using distilled water as a release medium were performed in this study. The water uptake of the gel beads at various times is shown in Figure 7. The PPN-loaded CA gel beads gave a rapid water absorption at 15 min and then reduced at 30 and 60 min of the test. The PPN-loaded CA-QPM gel beads with various SA:QPM ratios provided a comparable water absorption and did not differ from the PPN-loaded CA beads at 30 and 60 min of the test. The PPN release profiles of the gel beads in distilled water are illustrated in Figure 8. The PPN-loaded CA beads gave the fastest drug release, whereas increasing QPM ratios could slow down the drug release. The PPN release parameters of the gel beads were analyzed and are listed in Table 3. The initial burst release at 2 min of the PPN-loaded CA beads was 37.2%, and this parameter decreased with increasing QPM ratios in the gel beads. The T_50%_ and T_75%_ values were remarkably higher with increasing QPM ratios in the gel beads, suggesting that the PPN release rate decreased when QPM was incorporated. The PPN release kinetics from the gel beads in distilled water were analyzed using the power law. The release exponent (*n*) and kinetic constant (*k*) values are listed in Table 3. The release exponent value of the PPN-loaded CA beads was 0.483, indicative of a Fickian diffusion mechanism. The addition of QPM into the gel beads caused an increase in the release exponent value in the range of 0.689–0.735, suggesting an anomalous transport of PPN.

The drug release pattern of the CA gel beads depended on the pH and cations in the release medium. Using 0.1 N HCl solution, the calcium ions in the CA gel beads were entirely exchanged with hydrogen ions, resulting in a unionized form of the carboxyl groups of SA. This reaction has led to the matrix occurrence of an insoluble alginic acid for drug release retardation. On the other hand, the CA gel beads could hydrate and swell by ion exchange between cross-linking calcium ions and sodium ions in a sodium ion-rich medium (neutral pH of phosphate buffer solution) [4]. Additionally, the CA gels could be solubilized in the phosphate ion-rich medium; the phosphate ions acted as a complexing agent of calcium ions at a pH higher than 5.5 [39]. These phenomena brought about a partial formation of a water-soluble SA. For these reasons, the CA gel beads displayed higher water absorption and swelling properties in a neutral pH phosphate buffer, and then the swollen gel beads disintegrated. Therefore, the gel beads’ drug release was controlled by swelling [23,40]. Cations could influence the matrix network structure of the CA gel beads in a release medium mentioned above; therefore, a non-ionic medium (distilled water) was employed in this study to stabilize the gel bead structure during testing. The effect of QPM added on the water uptake and drug release of the CA beads was pointed out in this study. The PPN released from the CA gel beads was controlled by drug diffusion through the matrix structure of the gel beads. The incorporation of QPM that electrostatically interacted with SA before cross-linking did not affect the water uptake of the gel beads. However, the PPN-loaded CA-QPM gel beads displayed a higher release exponent (*n*) than the PPN-loaded CA beads. Therefore, it could be described that the PPN release was involved by drug diffusion and swelling of the gel beads.

## 3. Conclusions

The SA-QPM composite dispersions can be prepared using simple mixing. The molecular interaction between SA molecules and QPM particles caused a flocculate formation that the SA-QPM flocculates present a larger particle size with negative charges. The SA-QPM composite dispersions have Newtonian flow behavior similar to the SA dispersions, but the viscosity enhancement is found in the composite dispersions. The CA-QPM gel beads loaded with PPN display more spherical than the PPN-loaded CA gel beads. Incorporating QPM into the CA beads results in a bigger particle size and higher % DEE, which depends on the QPM ratios. The PPN-loaded CA-QPM gel beads displayed remarkably higher particle strength than the PPN-loaded CA beads. Furthermore, increased QPM content led to a lower burst release and slower PPN release from the gel beads regardless of the similar water uptake property. However, the drug release from the PPN-loaded CA-QPM gel beads are involved by drug diffusion and swelling of the gel beads. This study suggests that QPM can modulate the rheological properties of the SA dispersions, and the CA-QPM gel beads present a good potential to use as a delivery system for bioactive compounds.

## 4. Materials and Methods

### 4.1. Materials

SA (mannuronic acid/guluronic acid ratio = 0.59, CAS no. 9005-38-3) was obtained from ISP Thailand Ltd. (Bangkok, Thailand). Aqueous dispersions of QPM (Eudragit^®^ RL 30D, CAS no. 33434-24-3) and PPN (CAS no. 318-98-9) were purchased from Röhm Pharma GmbH (Darmstadt, Germany) and Changzhou Yabang Pharmaceutical Co., Ltd. (Jiangsu, China), respectively. All other reagents used were of analytical grade and used as received.

### 4.2. Preparation of SA-QPM Composite Dispersions

SA (0.75 g) was dispersed in 30 mL of distilled water for a homogeneous dispersion. QPM dispersions (30 %*w*/*w* of solid content) in the weights of 0, 1.25, 2.5, or 5 g were dropped and blended into the SA dispersion to achieve the SA:QPM ratios of 1:0, 1:0.5, 1:1, or 1:2, respectively, by weight. Next, the final volume of the dispersions was adjusted to 50 mL. The composite dispersions were incubated at 37 °C in a shaking water bath (75 oscillate min^−1^) for 24 h before investigation.

### 4.3. Characterization of SA-QPM Composite Dispersions

#### 4.3.1. Microscopic Morphology Studies

The dispersed phase morphology in the dispersions was investigated using an inverted microscope (Eclipse TS100, Nikon, Tokyo, Japan) and imaged using a digital camera (Coolpix 4500, Nikon, Tokyo, Japan).

#### 4.3.2. Particle Size Determination

The particle sizes of the dispersed phase in the dispersions were determined using a laser diffraction analyzer (Mastersizer2000 Model Hydro2000SM, Malvern Instrument Ltd., Malvern, UK). A small volume sample dispersion unit with 70 mL of distilled water was used. The composite dispersions were dropped into the sample dispersion unit, and the agitation at a rate of 50 Hz was applied for 30 s before determination. Then, the particle size of the dispersed phase in terms of volume-weighted mean diameter was recorded.

#### 4.3.3. Zeta Potential Measurement

The zeta potential of the dispersed phase was measured using a laser Doppler electrophoresis analyzer (Zetasizer Model ZEN 2600, Malvern Instrument Ltd., Malvern, UK). The measurement was performed at 25 °C. Next, an appropriate concentration of the composite dispersions was prepared using ultrapure water to meet a count rate higher than 20,000 counts s^−1^. Finally, the values of the zeta potential were recorded.

#### 4.3.4. Rheological Studies of Composite Dispersions

Rheological properties and viscosity of the dispersions were determined using a small sample adapter and spindle no.34 of a Brookfield Digital Rheometer (Model DV-III, Brookfield Engineering Labs Inc., Stoughton, MA, USA) at 37 ± 1 °C. A rheogram, which was the relationship between shear rate and shear stress at different revolution rates, of the samples was plotted. The flow type of the samples was analyzed using the following exponential formula [41]:

$$F^{N}=\eta G$$

logG=N log F−log η

*G*, *F*, *N*, and *η* are shear rate, shear stress, exponential constant, and viscosity coefficient, respectively. When the *N* value was close to unity, the rheological properties of the dispersions were Newtonian flow. If the *N* value was higher or lower than unity, the pseudoplastic or dilatant flow of the dispersions was obtained, respectively. Moreover, the viscosity of the dispersions at a constant shear rate was computed and used for comparison.

### 4.4. Preparation of Calcium Ion Cross-Linked Gel Beads

SA (1.5 g) was dispersed in 40 mL of distilled water for homogeneous dispersion. QPM dispersions (30 %*w*/*w* of solid content) in the weights of 0, 2.25, 5, and 10 g were dropped and blended into the SA dispersion to achieve the SA:QPM ratios of 1:0, 1:0.5, 1:1, or 1:2, respectively, by weight. PPN solution in the concentration of 1 %*w*/*v* was prepared using distilled water. The 25 mL of 1 %*w*/*v* PPN solution was continuously dropped and mixed into the SA-QPM composite dispersions. The final volume of the PPN-loaded SA-QPM dispersion was adjusted to 100 mL using distilled water. The composite dispersions with PPN were incubated at 37 °C in a shaking water bath (75 oscillate min^−1^) for 24 h. The cross-linked gel beads were prepared by dropping the PPN-loaded SA-QPM dispersions into 160 mL of 2.0 %*w/v* calcium chloride solution using a syringe with a 1.2-mm inner diameter needle. The PPN-loaded CA-QPM gel beads were treated in this solution with gentle stirring for 30 min, washed using 20 mL of distilled water twice, removed excess water using filter paper, and dried at 50 °C for one day. Before testing, the dried PPN-loaded CA-QPM gel beads were stored in a silica gel bead desiccator.

### 4.5. Characterization of PPN-Loaded CA-QPM Gel Beads

#### 4.5.1. Particle Size and Morphology Studies

The particle size of the gel beads was determined using an optical microscope (Olympus CH300RF200, Olympus Optical Co., Ltd., Tokyo, Japan). One hundred fifty beads were randomly selected, and their Feret diameters were measured. Moreover, the particle morphology of the gel beads was investigated using scanning electron microscopy (SEM). Finally, the gel beads were fixed onto stubs using double-sided adhesive tape and coated with gold in a vacuum evaporator. The morphology of the gel beads was observed and viewed using a scanning electron microscope (LEO1450VP, LEO Electron Microscopy Ltd., Cambridge, UK).

#### 4.5.2. Determination of Drug Entrapment Efficiency (DEE)

The ground gel beads in the weight of 0.1 g were immersed in 50 mL of distilled water. The mixture was stirred and sonicated for 15 min and treated at 37 °C for 6 h. Next, the supernatant was filtered using a cellulose acetate membrane (0.45-mm pore size), and the concentration of PPN was analyzed using a UV-vis spectrophotometer (Shimadzu UV1201, Kyoto, Japan) at a wavelength of 289 nm. The PPN content in the gel beads was computed in the unit of %*w*/*w*. Furthermore, the DEE (%) was calculated according to the beads’ actual to-theoretical drug content ratio [16,18,23].

#### 4.5.3. Mechanical Property of Gel Beads

The gel beads’ mechanical properties were studied using the modified method from the previously reported method [25,42]. A texture analyzer (TA.XT plus, Stable Micro Systems, Godalming, UK) with a 50-kg load cell connected with a 6-mm diameter cylindrical probe was employed. One gel bead was placed on the platform, then the probe was moved down and positioned to touch the gel bead. The initial distance was recorded, and the probe flattened the bead at a constant speed of 1.0 mm s^−1^. After that, the probe was stopped and removed when the gel bead was pressed to 50% of its original height. The relationship between force and percent displacement was plotted, and the maximum force at 50% displacement, which displays the particle strength of the gel beads, was recorded.

#### 4.5.4. Water Uptake Measurement

PPN-loaded SA-QPM gel beads (50 mg) were placed in a small basket. The basket was immersed in a beaker containing 50 mL of distilled water. The temperature of distilled water was controlled at 37 ± 1 °C, and the beaker was shaken occasionally. After a predetermined time, the basket was withdrawn, removed the remaining water, and immediately weighed on an analytical balance [23,39]. The water uptake of the gel beads can be computed using the following equation:

$$Water\;uptake\;\left(\%\right)=\left(\frac{W_{t}-W_{0}}{W_{0}}\right)\times 100$$

where *W_t_* and *W*_0_ are the wet and initial mass of beads, respectively.

#### 4.5.5. In Vitro Drug Release Studies

The PPN-loaded CA-QPM gel beads (100 mg) were suspended in 30 mL of distilled water in a polypropylene test tube that was incubated at 37 °C in a shaking water bath at a rate of (75 oscillate min^−1^. Samples (3 mL) were collected at the predetermined time intervals and replaced with 3 mL of distilled water, maintaining a constant release volume. The concentration of PPN released was analyzed spectrophotometrically at the wavelength of 289 nm using a UV-visible spectrophotometer (Shimadzu UV1201, Kyoto, Japan). The release data obtained, T_50%_ and T_75%_, the times required to achieve 50% and 75% drug content were calculated and used for comparison.

The PPN release kinetics from the gel beads in distilled water were investigated by fitting the PPN release data into the power law, which can be expressed using the following equation:

$$\frac{M_{t}}{M_{\infty }}=kt^{n}$$

$$log\frac{M_{t}}{M_{\infty }}=n\log t+\log k$$

where *M_t_/M_∞_*, *k*, and *n* are the fractional drug release at time t, the kinetic constant, and the release exponent indicative of the drug release mechanism, respectively. A release exponent of n = 0.5 corresponds to a diffusion-controlled drug release (Fickian diffusion), whereas a release exponent of *n* = 1 indicates a polymer swelling/erosion-controlled release mechanism. Thus, release exponents between these two extreme values indicate an anomalous transport, a mixing of drug diffusion, and swelling/erosion of polymer [43].

## Figures and Tables

**Table 1:** Rheological property and viscosity of SA and SA-QPM dispersions.

Dispersions	Exponential Constant, *N*	Viscosity Coefficient, *η* ((dyne cm^−2^)*^N^* s)	Viscosity at 22.4 s^−1^ Shear Rate (cP)
SA:QPM ratios			
1:0	1.00 ± 0.01	2.70 ± 0.08	270.39 ± 1.12
1:0.5	0.95 ± 0.02	3.80 ± 0.20	326.16 ± 1.86
1:1	0.98 ± 0.01	3.07 ± 0.11	289.58 ± 0.52
1:2	0.94 ± 0.01	3.64 ± 0.08	311.76 ± 1.80

Data are mean ± S.D., *n* = 3.

**Table 2:** Particle size and drug entrapment efficiency of PPN-loaded CA-QPM gel beads.

Gel Beads	Particle Size ^a^ (mm)	Drug Content ^b^ (%*w*/*w*)	DEE ^b^ (%)
SA:QPM ratios			
1:0	1.03 ± 0.12	3.27 ± 0.06	13.10 ± 0.25
1:0.5	1.17 ± 0.13	2.76 ± 0.10	15.17 ± 0.56
1:1	1.29 ± 0.18	2.64 ± 0.01	18.49 ± 0.07
1:2	1.33 ± 0.14	2.29 ± 0.02	22.82 ± 0.25

^a^ Data are mean ± S.D., *n* = 150; ^b^ Data are mean ± S.D., *n* = 3.

**Table 3:** Drug release characteristics of PPN-loaded CA-QPM gel beads.

Gel Beads	Burst Release ^a^ (%)	T_50%_^a^ (min)	T_75%_^a^ (min)	Release Exponent, *n*	Kinetic Constant, *k*
SA:QPM ratios					
1:0	37.23 ± 5.63	3.41 ± 0.52	8.34 ± 0.74	0.483	0.274
1:0.5	10.01 ± 3.01	11.94 ± 2.52	26.10 ± 3.97	0.689	0.089
1:1	13.15 ± 0.58	12.49 ± 0.55	30.67 ± 0.72	0.694	0.086
1:2	9.50 ± 1.56	19.70 ± 0.82	49.02 ± 2.39	0.735	0.058

^a^ Data are mean ± S.D., *n* = 3.

## Data Availability

Data are contained within the article.
